# Allelochemicals from the Rhizosphere Soil of Potato (*Solanum tuberosum* L.) and Their Interactions with the Soilborne Pathogens

**DOI:** 10.3390/plants11151934

**Published:** 2022-07-26

**Authors:** Aiyi Xin, Hui Jin, Xiaoyan Yang, Jinfeng Guan, Heping Hui, Haoyue Liu, Zengtuan Cui, Zhiheng Dun, Bo Qin

**Affiliations:** 1 CAS Key Laboratory of Chemistry of Northwestern Plant Resources and Key Laboratory for Natural Medicine of Gansu Province, Lanzhou Institute of Chemical Physics, Chinese Academy of Sciences (CAS), Lanzhou 730000, China; cpuxay@163.com (A.X.); comefine@licp.cas.cn (H.J.); yangxiaoy@licp.cas.cn (X.Y.); mysy105@163.com (H.H.); liuhaoyue0820@163.com (H.L.); 2 School of Chemistry and Chemical Engineering, Mianyang Normal University, Mianyang 621000, China; 3 Institute for Food and Drug Control, Tongliao City, Inner Mongolia Autonomous Region, Tongliao 028000, China; nmtlgjf@sina.com; 4 Cultivated Land Quality Construction and Management Station of Gansu Province, Lanzhou 730030, China; konphy@126.com (Z.C.); ruoxi020xw@163.com (Z.D.)

**Keywords:** potato (*Solanum tuberosum* L.), replant problems, allelochemicals, autotoxicity, antifungal effects

## Abstract

To reveal the allelopathic effects of potato, seven compounds were isolated from the rhizosphere soil: 7-methoxycoumarin (**1**), palmitic acid (**2**), caffeic acid (**3**), chlorogenic acid (**4**), quercetin dehydrate (**5**), quercitrin (**6**), and rutin (**7**). Bioassays showed that compounds **1**, **2**, **4**, and **6** had inhibitory effects on the growth of *L. sativa* and tissue culture seedlings of potato. The existence of the allelochemicals was confirmed by HPLC, and their contents were quantified with a total concentration of 9.02 μg/g in the rhizosphere soil of replanted potato. Approaches on the interactions of the allelochemicals and pathogens of potato including *A. solani*, *B. cinerea*, *F. solani*, *F. oxysporum*, *C. coccodes*, and *V. dahlia* revealed that compound **1** had inhibitory effects but compounds **2**–**4** promoted the colony growth of the pathogens. These findings demonstrated that the autotoxic allelopathy and enhancement of the pathogens caused by the accumulation of the allelochemicals in the continuously cropped soil should be one of the main reasons for the replant problems of potato.

## 1. Introduction

Tubers of potato (*Solanum tuberosum* L.) are the third most important food crops in terms of human consumption with a population of more than a billion people worldwide consuming them. The global total crop production exceeds 374 million metric tons each year, following wheat (*Triticum aestivum* L.), rice (*Oryza sativa* L.), and maize (*Zea mays* L.) according to the statistics data of the Food and Agriculture Organization of the United Nations (FAO, Rome, Italy) in 2017 [1,2]. Being rich in starch and crude protein, potato is used for both a staple food and a vegetable. Research indicated that potato can provide a great diversity of nutrients such as protein, lipids, minerals, vitamins, and dietary fiber [3]. Owing to the high content of essential amino acids, potato protein is considered as one of the most valuable non-animal proteins [4]. Meanwhile, it is a typical highly productive crop and has been used as a source of starch and alcohol for industrial uses; thus the production of potato has been regarded as a pillar industry for farmers to get rid of poverty and increase income in some major producing areas [5,6].

However, the crop production of potato has often been hampered by replant problems. The occurrence of replant failure not only affects the yield and quality of potato, but also reduces the safety of the product [7]. Therefore, it is urgent to declare the reasons causing the replant failure and explore an efficient way to achieve high and stable production of potato. Replant failure is a phenomenon in which several factors are involved; allelopathy (especially autotoxic allelopathy) and the enhancement of soilborne pathogens coupled with an imbalance in the soil microbial community caused by allelochemicals have been considered as essential factors on this issue [8,9,10,11]. A great variety of secondary metabolites produced by the crop and released into the soil environment through leaching, root exudation, and/or residue decomposition have been shown to interact with the replanted plant itself and other organisms [12,13,14,15,16]. Further knowledge indicated that soil-borne pathogens play a primary role in the occurrence of replant disease [17]. It is proposed that the activities of soil-borne pathogens would be improved indirectly by the allelochemicals which are released by the crop [18]. The allelochemicals are not only autotoxic compounds for the replanted plant, but also a precipitating factor for changing the composition of the microbial community to lead to the build-up of soil-borne pathogens [19,20]. The accumulation of phytotoxic substances and the decrease in bacterial diversity is attributed to replant failure which was occurred during continuous cropping [21]. Several common diseases of potato were caused by the fungi in major producing regions around the world, such as *Alternaria solani* that may result in early blight, *Botrytis cinerea* that may bring about gray mold, *Fusarium solani* that may increase the risk of dry rot, *Fusarium oxysporum* that may cause root rot, *Colletotrichum coccodes* that may lead to potato black dot, *Verticillium dahliae* that may give rise to potato early dying, etc.

It was reported that water extracts from different organs and root exudates of potato had potential phytotoxicity against potato itself and other plants [22,23,24,25]. However, the specific compounds which are responsible for the phytotoxicity still remain unknown, and the relative roles of autotoxic factors and pathogens of potato are unclear as well. Elucidating the chemical reasons for the replant problem is necessary to explor efficient approaches to eliminating the replant failure of potato. Thus, in this work, the allelochemicals from the rhizosphere soil of cultivated potato were isolated and identified for the first time. Further investigations on the interactions of the allelochemicals and pathogens were carried out as well to get more helpful information to understand the chemical basis of the replant failure of potato.

## 2. Results

### 2.1. Allelopathic Activity of the Crude Extract of the Rhizosphere Soil of Potato

**Figure 1:**
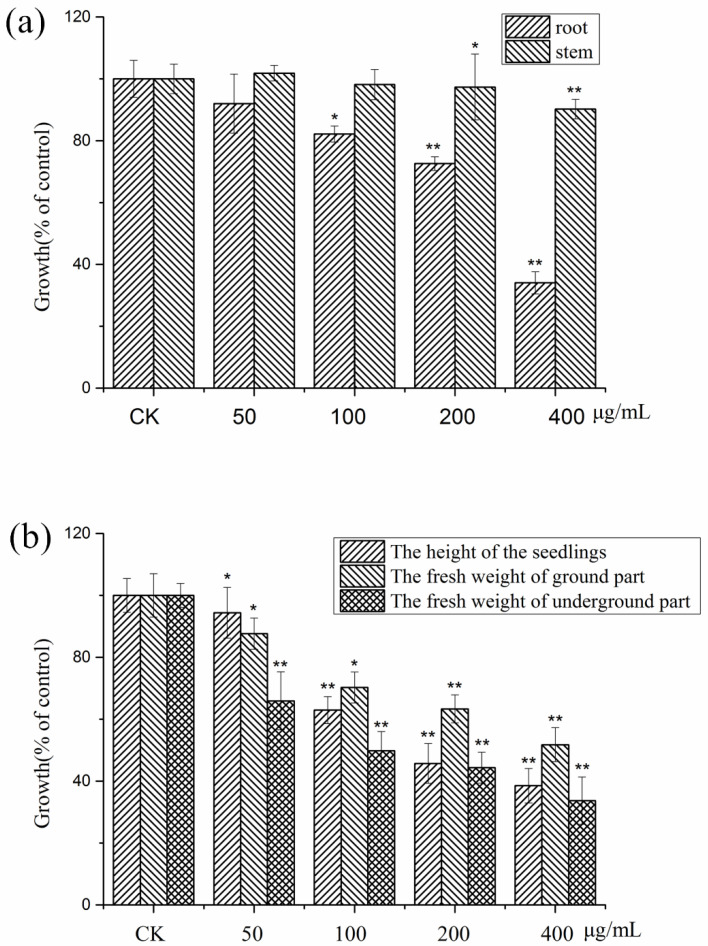
Phytotoxic effects of the crude extract of the rhizosphere soil of potato on seedlings of *L. sativa* (**a**) and potato (**b**) at concentrations of 50, 100, 200, and 400 μg/mL, respectively. Values are presented as a percentage of the mean compared to the control. Means significantly lower than the methanol controls are indicated by * (one way ANOVA; *p* < 0.05) or ** (*p* < 0.01). Error bars are one standard error of the mean. *n* = 3.

The phytotoxic activity of the crude extract was evaluated with *Lactuca sativa* L. (Figure 1a) and potato tissue culture (Figure 1b) seedlings. The results revealed that the crude methanol extract significantly inhibited the growth of the treated seedlings and the effect was in a concentration-dependent manner. The crude extract exhibited a more remarkable inhibitory effect on the growth of roots than that on stems for *L. sativa* seedlings. As Figure 1a showed, the inhibition ratios of roots at the treated concentrations of 100, 200, and 400 μg/mL were 17.82%, 27.39%, and 65.96%, respectively. The growth of potato seedlings was observably inhibited by the crude extract in a similar manner. The inhibitory effect on the growth of the roots was more pronounced than that on the leaves and stems for the potato seedlings, which was in accordance with the growth of *L. sativa*. As shown in Figure 1b, at a concentration of 100 μg/mL, the inhibition ratios of the height of the potato seedlings, the fresh weight of leave and stems, and the fresh weight of the roots were 37.03%, 29.73%, and 50.17%, respectively. The inhibition ratios at the treated concentration of 200 μg/mL reached up to 54.25%, 36.68%, and 55.63%, respectively. At the maximum concentration (400 μg/mL), the inhibition ratios ran up to 61.26%, 48.20%, and 66.28%, respectively.

### 2.2. Isolation of Allelochemicals from the Rhizosphere Soil of Potato

**Figure 2:**
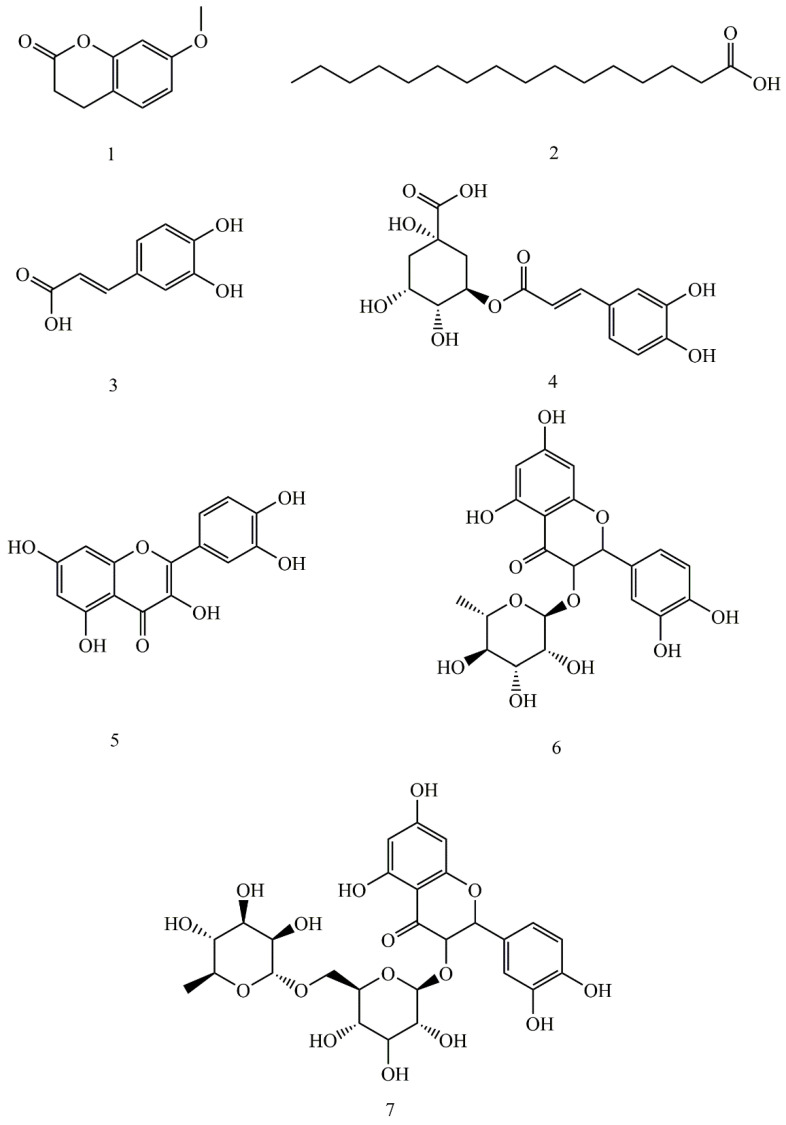
Structures of compounds **1**–**7** isolated from the rhizosphere soil of potato. (**1**. 7-methoxycoumarin, **2**. palmitic acid, **3**. caffeic acid, **4**. chlorogenic acid, **5**. quercetin dehydrate, **6**. quercitrin, and **7**. rutin).

Seven compounds were isolated from the extract of the rhizosphere soil of potato; they were identified as: 7-methoxycoumarin (**1**), palmitic acid (**2**), caffeic acid (**3**), chlorogenic acid (**4**), quercetin dehydrate (**5**), quercitrin (**6**), and rutin (**7**). Their structures were characterized by spectroscopic analysis in comparison with literature data. The structures of compounds **1**–**7** are shown in Figure 2.

Compound **1**: C_10_H_8_O_3_

MS: [M + Na]^+^ C_10_H_8_NaO_3_, measured m/z 199.0363, calculated m/z 199.0371, err 3.9 ppm.

^1^H NMR (400 MHz, CD_3_OD) δ 7.65 (d, J = 9.5 Hz, 1H), 7.38 (d, J = 8.6 Hz, 1H), 6.85 (dd, J = 8.6, 2.3 Hz, 1H), 6.80 (d, J = 2.1 Hz, 1H), 6.25 (d, J = 9.5 Hz, 1H).

^13^C NMR (100 MHz, CD_3_OD) δ 162.57, 160.94, 155.63, 143.21, 128.54, 112.79, 112.30, 100.57, 55.52.

Compound **2**: C_16_H_32_O_2_

MS: [M + Na]^+^ C_16_H_32_NaO_2_, measured m/z 279.2295, calculated m/z 279.2300, err 1.8 ppm.

^1^H NMR (400 MHz, CDCl_3_) δ 2.34 (t, J = 7.5 Hz, 2H), 1.66-1.60 (m, 2H), 1.26 (s, 24H), 0.88 (t, J = 6.7 Hz, 3H).

^13^C NMR (100 MHz, CDCl_3_) δ 180.57, 34.13, 31.94, 29.71, 29.70, 29.68, 29.67, 29.66, 29.61, 29.45, 29.38, 29.25, 29.08, 24.68, 22.70, 14.10.

Compound **3**: C_9_H_8_O_4_

MS: [M + Na]^+^ C_9_H_8_NaO_4_, measured m/z 203.0310, calculated m/z 203.0315, err 2.5 ppm.

^1^H NMR (400 MHz, CD_3_OD) δ 7.53 (d, J = 15.9 Hz, 1H), 7.03 (d, J = 1.9 Hz, 1H), 6.92 (dd, J = 8.2, 1.9 Hz, 1H), 6.77 (d, J = 8.2 Hz, 1H), 6.21 (d, J = 15.9 Hz, 1H).

^13^C NMR (100 MHz, CD_3_OD) δ 169.68, 148.06, 145.69, 145.39, 126.41, 121.50, 115.11, 114.11, 113.71.

Compound **4**: C_16_H_18_O_9_

MS: [M + Na]^+^ C_16_H_18_NaO_9_, measured m/z 377.0840, calculated m/z 377.0843, err 0.8 ppm.

^1^H NMR (400 MHz, Acetone) δ 7.56 (d, J = 15.9 Hz, 1H), 7.16 (d, J = 1.9 Hz, 1H), 7.05 (dd, J = 8.2, 1.9 Hz, 1H), 6.88 (d, J = 8.2 Hz, 1H), 6.27 (d, J = 15.9 Hz, 1H), 5.42–5.36 (m, 1H), 4.25 (d, J = 3.3 Hz, 1H), 3.78 (d, J = 3.0 Hz, 1H), 2.29–2.22 (m, 1H), 2.16 (d, J = 3.1 Hz, 1H), 2.05 (d, J = 4.4 Hz, 2H).

^13^C NMR (100 MHz, Acetone) δ 174.20, 166.24, 147.86, 145.42, 144.88, 126.79, 121.65, 115.51, 115.00, 114.34, 75.36, 72.64, 70.76, 70.47, 38.26, 37.03.

Compound **5**: C_15_H_10_O_7_

MS: [M + Na]^+^ C_15_H_10_NaO_7_, measured m/z 325.0317, calculated m/z 325.0319, err 0.6 ppm.

^1^H NMR (400 MHz, CD_3_OD) δ 7.71 (d, J = 2.0 Hz, 1H), 7.61 (dd, J = 8.5, 2.1 Hz, 1H), 6.86 (d, J = 8.5 Hz, 1H), 6.36 (d, J = 2.0 Hz, 1H), 6.15 (d, J = 2.0 Hz, 1H).

^13^C NMR (100 MHz, CD_3_OD) δ 177.33, 165.57, 162.51, 158.22, 148.76, 147.97, 146.22, 137.24, 124.14, 121.66, 116.21, 115.97, 104.51, 99.21, 94.39.

Compound **6**: C_21_H_20_O_11_

MS: [M + Na]^+^ C_21_H_20_NaO_11_, measured m/z 47.0908, calculated m/z 471.0898, err −2.1 ppm.

^1^H NMR (400 MHz, CD_3_OD) δ 7.34 (d, J = 1.9 Hz, 1H), 7.32 (dd, J = 8.3, 2.0 Hz, 1H), 6.92 (d, J = 8.3 Hz, 1H), 6.38 (d, J = 2.0 Hz, 1H), 6.21 (d, J = 2.0 Hz, 1H), 5.36 (s, 1H), 4.22 (d, J = 1.5 Hz, 1H), 3.75 (dd, J = 9.3, 3.3 Hz, 1H), 3.43 (dd, J = 9.5, 6.1 Hz, 1H), 3.35 (s, 1H), 0.95 (d, J = 6.1 Hz, 3H).

^13^C NMR (100 MHz, CD_3_OD) δ 179.45, 165.69, 163.03, 159.11, 158.33, 149.60, 146.22, 136.03, 122.76, 122.64, 116.71, 116.16, 105.69, 103.34, 99.60, 94.49, 73.04, 71.91, 71.83, 71.70, 17.44.

Compound **7**: C_27_H_30_O_16_

MS: [M + Na]^+^ C_27_H_30_NaO_16_, measured m/z 633.1435, calculated m/z 633.1426, err −1.5 ppm.

^1^H NMR (400 MHz, CD_3_OD) δ 7.66 (s, 1H), 7.62 (d, J = 8.4 Hz, 1H), 6.86 (d, J = 8.4 Hz, 1H), 6.37 (d, J = 1.9 Hz, 1H), 6.18 (d, J = 1.9 Hz, 1H), 5.10 (d, J = 7.4 Hz, 1H), 4.52 (s, 1H), 3.80 (d, J = 10.5 Hz, 1H), 3.64 (d, J = 1.5 Hz, 1H), 3.54 (dd, J = 9.5, 3.3 Hz, 1H), 3.48 (d, J = 9.1 Hz, 1H), 3.45 (d, J = 5.1 Hz, 2H), 3.40 (d, J = 4.8 Hz, 1H), 3.36 (d, J = 4.5 Hz, 1H), 3.33 (s, 1H), 3.27 (d, J = 3.2 Hz, 1H), 1.12 (d, J = 6.2 Hz, 3H).

^13^C NMR (100 MHz, CD_3_OD) δ 177.98, 164.58, 161.53, 157.92, 157.07, 148.39, 144.41, 134.26, 129.05, 122.18, 121.72, 116.32, 114.66, 104.22, 103.37, 101.02, 98.55, 93.48, 76.78, 75.79, 74.34, 72.55, 70.70, 69.99, 68.31, 67.16, 16.50.

### 2.3. Allelopathic Activities of the Purified Compounds on L. sativa Seedlings

**Figure 3:**
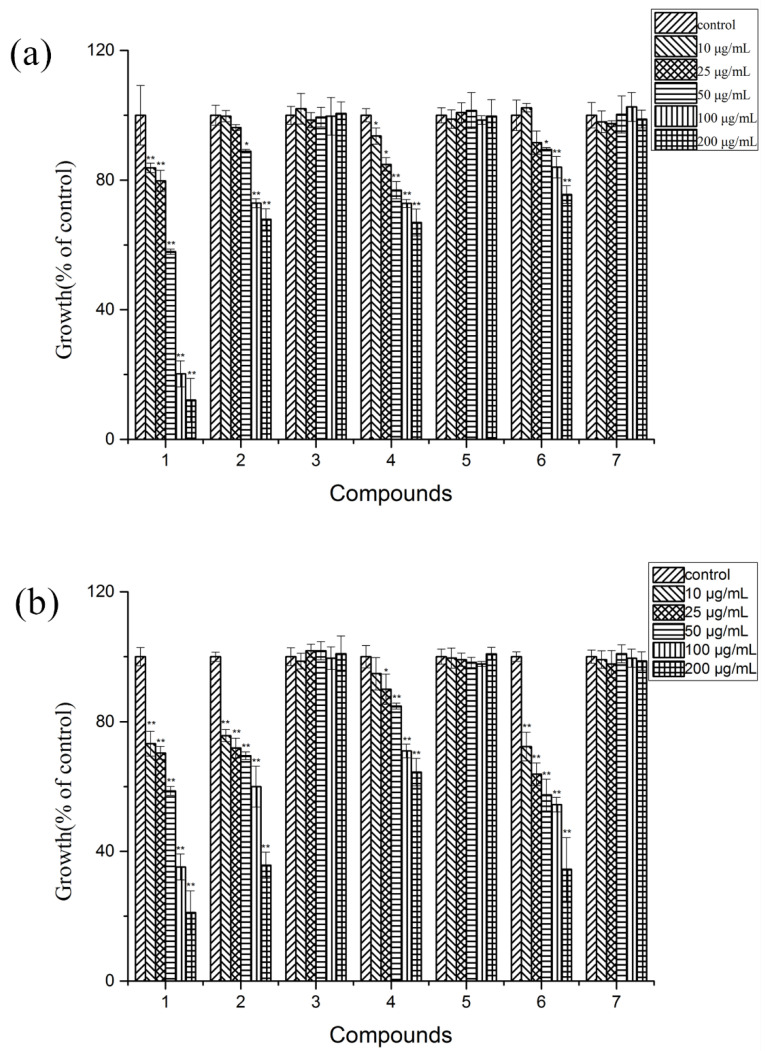
Phytotoxic effects of compounds **1**–**7** on root (**a**) and stems (**b**) lengths of *L. sativa* seedlings at concentrations of 10, 25, 50, 100, and 200 μg/mL, respectively. Values are presented as a percentage of the mean compared to the control. Means significantly lower than the DMSO controls are indicated by * (one way ANOVA; *p* < 0.05) or ** (*p* < 0.01). Error bars are one standard error of the mean. *n* = 3.

As shown in Figure 3, compounds **1**, **2**, **4**, and **6** inhibited the growth of the seedlings of *L. sativa*, while compounds **3**, **5**, and **7** had no effect. The inhibitory effect increased with the increase in treated concentrations. Compound **1** displayed a strong inhibition on the growth of *L. sativa* at all treated concentrations. The inhibition ratios to the roots and stems of *L. sativa* at the concentration of 200 μg/mL were 78.87% and 87.87%, respectively. Compounds **2**, **4**, and **6** showed strong inhibition on the growth of *L. sativa* at higher concentrations (≥50 μg/mL), while there were slight or no effects at low treated concentrations. Meanwhile, the inhibition ratios on roots were more remarkable than that on stems for *L. sativa* seedlings. At the maximum concentration (200 μg/mL), the inhibition ratios of roots reached to 64.29%, 35.50%, and 65.53%, respectively.

### 2.4. Autotoxic Activities of the Purified Compounds on Tissue Culture Seedlings of Potato

**Figure 4:**
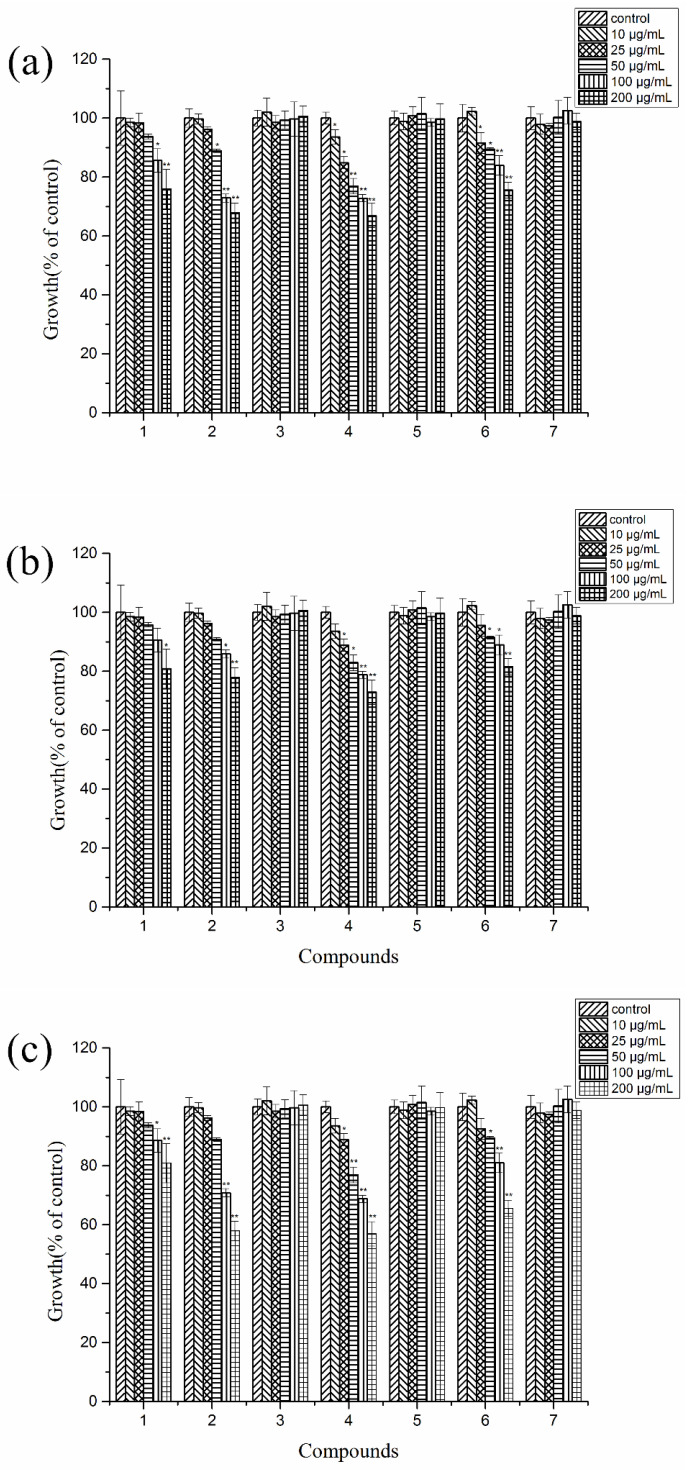
Autotoxic effects of compounds **1**–**7** on the height of the seedlings (**a**), the fresh weight of the leaves and stems (**b**), and the fresh weight of the roots (**c**) at concentrations of 10, 25, 50, 100, and 200 μg/mL, respectively. Values are presented as a percentage of the mean compared to the control. Means significantly lower than the DMSO controls are indicated by * (one way ANOVA; *p* < 0.05) or ** (*p* < 0.01). Error bars are one standard error of the mean. *n* = 3.

**Figure 5:**
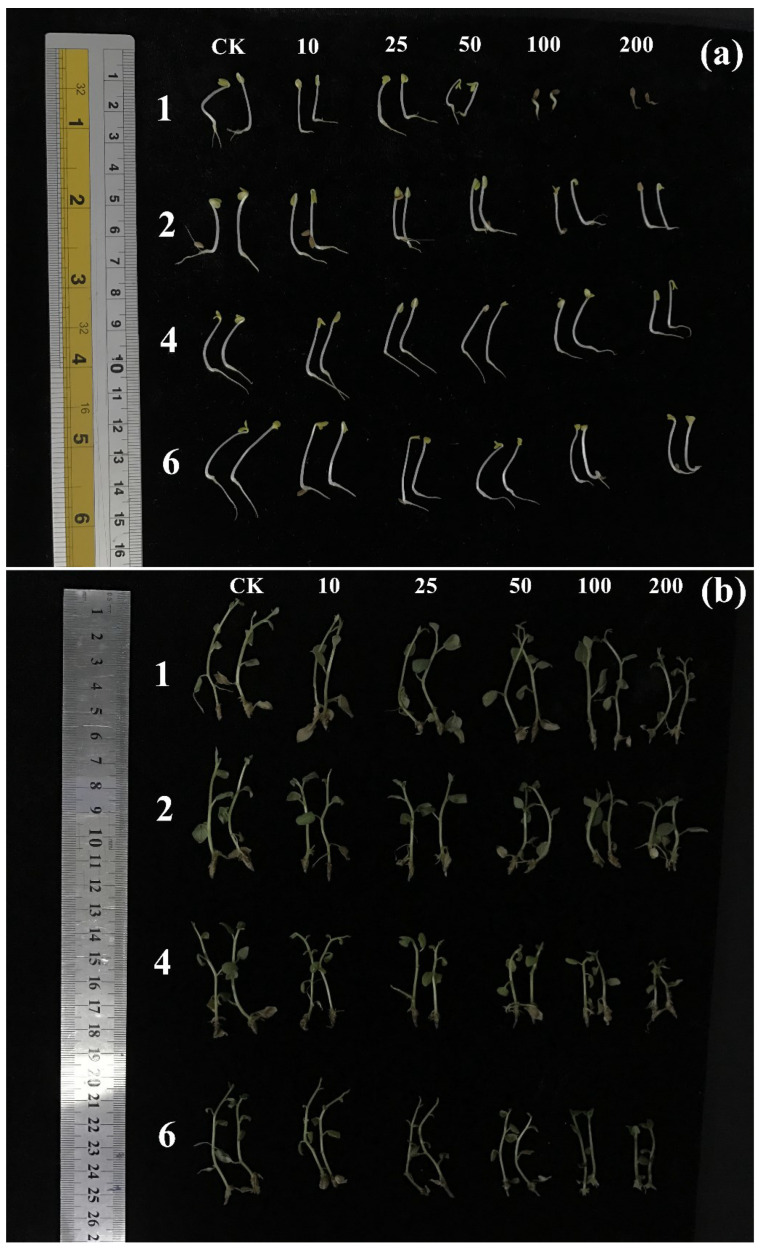
Typical pictures of the effects of compounds **1**, **2**, **4** and **6** on the growth of *L. sativa* seedlings (**a**) and potato tissue culture seedlings (**b**) at concentrations of 0, 10, 25, 50, 100, and 200 μg/mL.

The autotoxic activity of the purified compounds was evaluated with tissue culture seedlings of potato. The results revealed that compounds **1**, **2**, **4**, and **6** had an inhibition effect on the growth of the potato seedlings, while compounds **3**, **5**, and **7** had no effect (Figure 4). Compound **1** showed an inhibitory effect on the growth of tissue culture seedlings of potato at the highest treated concentration (200 μg/mL), while there were no effects at lower concentrations. The inhibition ratios at the concentration of 200 μg/mL on the height of the seedlings, the fresh weight of the leaves and stems, and the fresh weight of the roots were 14.06%, 12.67%, and 20.17%, respectively. At higher treated concentrations (≥50 μg/mL), compounds **2**, **4**, and **6** displayed moderate inhibitions on the growth of potato tissue culture seedlings, while there were slight or no effects at lower treated concentrations. Similarly, the autotoxic effects on the fresh weight of the roots of tissue seedlings were more distinguished in a concentration-dependent manner. The inhibition ratios of the fresh weight of the leaves and stems at the maximum concentration (200 μg/mL) reached up to 42.07%, 34.41%, and 43.08%, respectively. The typical patterns of the effects of compounds **1**, **2**, **4**, and **6** on the growth length of *L. sativa* seedlings (a) and potato tissue culture seedlings (b) at concentrations of 0, 10, 25, 50, 100, and 200 μg/mL are shown in Figure 5.

### 2.5. Confirmation and Quantification of the Allelochemicals in the Rhizosphere Soil

**Figure 6:**
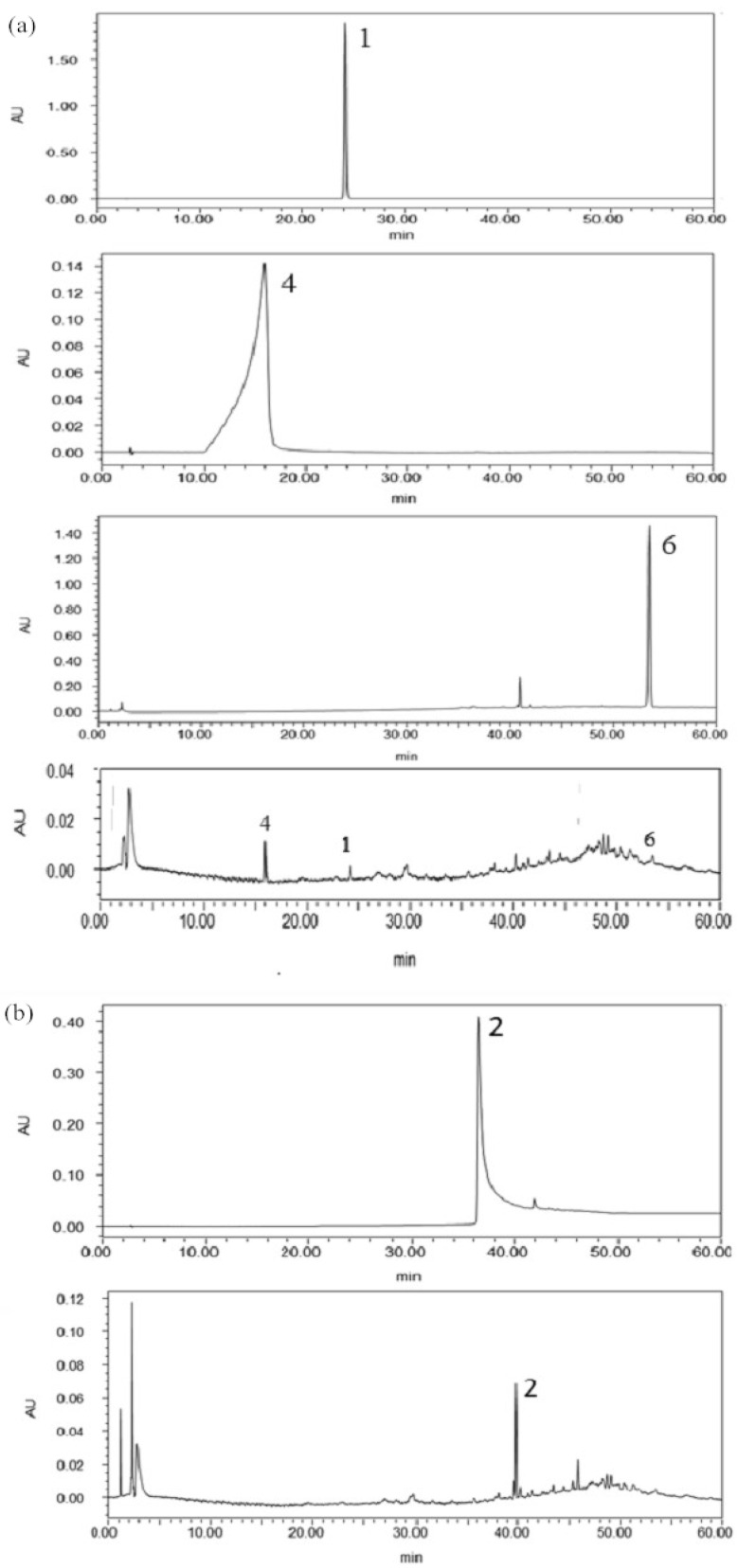
HPLC analysis of the allelochemicals in the rhizosphere soil of continuously cropped potato at 240 nm (**a**) and 320 nm (**b**), respectively; purified compounds **1**, **4**, and **6** were detected at 240 nm (**a**) and compound **2** was detected at 320 nm (**b**).

High-performance liquid chromatography (HPLC) was performed to quantify the allelochemicals in the rhizosphere soil of replanted potato. The presence of the compounds in the soil was confirmed by comparing the retention times of the standard compounds **1**, **2**, **4**, and **6** under the same chromatographic conditions (Figure 6). At the same time, the standard curve of the content of compounds **1**, **2**, **4**, and **6** was determined at a wavelength of 240 nm (compounds **1**, **2**, and **4**) and 280 nm (compound **6**) (Table 1). The contents of compounds **1**, **2**, **4**, and **6** in the rhizosphere soil were calculated to be 1.46, 6.64, 0.35, and 0.57 μg/g, respectively.

### 2.6. Antifungal Activity on Potato Pathogens of the Allelochemicals

**Figure 7:**
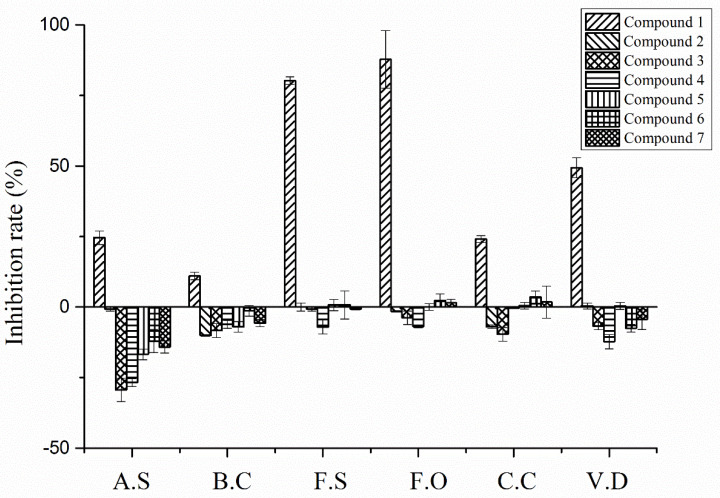
The effects of compounds **1**–**7** against the potato pathogens including *A. solani*, *B. cinerea*, *F. solani*, *F. oxysporum*, *C. coccodes*, and *V. dahlia* at a concentration of 200 μg/mL.

**Figure 8:**
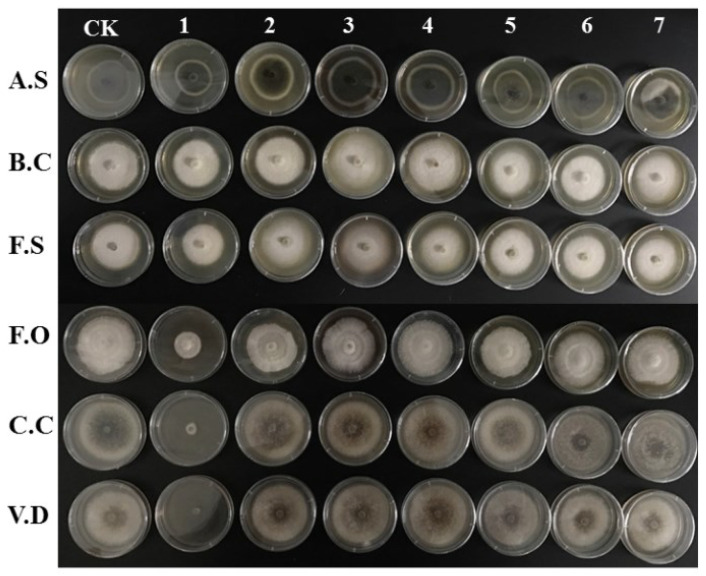
Typical pictures of the effects of compounds **1**–**7** against potato pathogens including *A. solani*, *B. cinerea*, *F. solani*, *F. oxysporum*, *C. coccodes*, and *V. dahlia* at a concentration of 200 μg/mL.

Compounds **1**–**7** showed different antifungal activity at a treated concentration of 200 μg/mL against pathogens of potato, including *A. solani*, *B. cinerea*, *F. solani*, *F. oxysporum*, *C. coccodes* and *V. dahlia*, as shown in Figure 7 and Figure 8. The results indicated that compounds **2**–**4** promoted the growth of the colonies of the six pathogens of potato; only compound **1** showed an inhibitory effect. The promotive indices of compound **2** on *A. solani*, *B. cinerea*, and *C. coccodes* at 200 μg/mL were 33.8%, 11.5%, and 7.7%, respectively. The promotive indices of compound **3** on *A. solani*, *B. cinerea*, *C. coccodes*, and *V. dahlia* at 200 μg/mL were 29.3%, 8.5%, 11.6%, and 7.2%, respectively. The promotive indices of compound **4** on *A. solani*, *B. cinerea*, *F. solani*, *F. oxysporum*, and *V. dahlia* at 200 μg/mL were 20.6%, 7.8%, 7.6%, 8.0%, and 14.9%, respectively.

## 3. Discussion

Negative plant–soil feedback is one of the important factors of the replant problem, which plays an essential role in hindering the sustainable development of agriculture [26]. Investigations indicated that the accumulation of allelochemicals in the soil environment is a crucial factor for the replant failure of large amounts of plants. The growth of replanted plants may be inhibited by the reduction in the number of meristem cells, the reduction in elongation zones, and the accumulation of auxins caused by allelochemicals [27].

The bioassay results of the crude extract in the present study indicated that there were potential allelochemicals in the rhizosphere soil of potato that contributed to the inhibitory effect on the seedling growth of itself and other plants. Nevertheless, the autotoxic effect of the allelochemicals of potato has not been previously clarified.

Hence, in this work, seven compounds were isolated from the rhizosphere soil of potato and bioassays indicated that compounds **1**, **2**, **4**, and **6** had inhibitory effects on the growth of *L. sativa* and potato tissue culture seedlings. Compound **1** belongs to coumarin, which is considered to be one kind of allelochemical by Rice [28]. It was reported that coumarin inhibited the seed germination and plant growth (length and mass of shoots and roots) of several plants [29,30]. The results in this study revealed that it had an inhibitory effect on the growth of lettuce seedlings and tissue culture seedlings of potato at the maximum treatment concentration. Compound **2** is in the range of fatty acids; investigations demonstrate that it has an enormous capacity of phytotoxic activity [31]. The results in this work showed that it inhibited the growth of lettuce seedlings and tissue culture seedlings of potato at high treated concentrations, which are consistent with the previous study. Chlorogenic acid (compound **3**) is abundant in potato peels. It has a broad range of therapeutic properties, including antioxidant activity andantibacterial, anti-inflammatory, and anti-obesity properties [32]. However, phenolic acids are one kind of important allelochemical as well, which generally exist in their cultivated soils [33]. It was reported that chlorogenic acid inhibited the growth of lettuce seedlings at high concentrations, and ROS in lettuce could be accumulated under the stress of chlorogenic acid [34]. The results in the present study indicated that the growth of seedlings of lettuce and potato were suppressed at a high treated concentration of compound **3**. Compound **6** was regarded as a flavonoid; it is reported that flavonoids affect many physiological functions of plants, including the inhibition of auxin transport, phytochemical defense, an allelopathic effect, and an antioxidant effect [35,36,37]. In the present study, compound **6** only displayed an inhibitory effect on the growth of the *L. sativa* and potato at the maximum treated concentration. Analysis of the characterized compounds in the rhizosphere soil showed that compound **2** had the highest content with a value of 6.64 μg/g, and the total content of the four autotoxic allelochemicals in the rhizosphere soil reached 9.02 μg/g.

Soil microorganisms are critical elements in agricultural ecosystems, and the compositions of soil microbial communities are influenced through rhizodeposition [38]. By this way, beneficial microbes were promoted, but also soil-borne pathogens were built-up [39,40]. The imbalance of soil microbial community has been considered as one of the essential factors for replant problems. Many cultivated plants, which suffer soil sickness, are often connected with soil-borne pathogens [41]. Several research studies suggested that replant disease could be induced directly by the occurrence of pathogens and indirectly by the release of toxic substances which were decomposed by plants [42,43]. Currently, allelochemicals are regarded as a significant cause which influences the microbial community [44]. The accumulation of allelochemicals brings about the increase in harmful pathogens in rhizosphere soil, leads to an unbalance of the structure of the microbial community, and results in replant diseases during continuous cropping [45]. In this work, the antifungal assays showed that compound **1** had an inhibitory effect on the six tested pathogens of potato, but compounds **2**–**4** promoted the colony growth of the pathogens, suggesting that the release of the autotoxic allelochemicals induced the disequilibrium of the microbial community in the continuously cropped potato soil. The compositions of the allelochemicals affected the composition of the microbial community in the replanted soil and enhanced the feasibility of the outbreak of the soil-borne diseases of potato during continuous cultivation.

## 4. Materials and Methods

### 4.1. General Experimental Instruments and Reagents

MCI gel CHP20/P120 (Mitsubishi chemical corporation, Tokyo, Japan) and silica gel 60 (200–300 mesh, Qingdao Haiyang Chemical Co., Ltd., Qingdao, China) were used for Column Chromatography (CC). Thin-layer chromatography (TLC) and preparative thin-layer chromatography were carried out on silica gel F254 plates, and the spots were detected under UV detection or by heating after spraying with 5% H_2_SO_4_ in C_2_H_5_OH (*v*/*v*). By using a Bruker Avance III-400 spectrometer (Bruker AXS GmbH, Karlsruhe, Germany), ^1^H and ^13^C NMR spectra (400 and 100 MHz, respectively) were recorded. Electrospray ionization quadruple time -of-flight mass spectrometry (ESI-Q-TOF MS) was used to obtain the high-resolution mass data.

The High-Performance Liquid Chromatographic analysis was conducted on a Waters 1525 binary HPLC pump (Waters, Milford, MA, USA) with a Waters 2998 photodiode array detector, coupled with a Waters C18 column (250 mm × 4.6 mm, 5 μm).

### 4.2. Soil Samples

Soil samples around the roots of potato cultivar Long 7 (within 5 cm) were collected in five different areas of Lanzhou, Gansu Province, China, in October 2018. The collected soil samples were dried in the dark at room temperature and passed through a 1 mm screen sieve. Then, the soil samples were extracted three times (90 min each) in methanol with ultrasonic treatments, and the filtrate was concentrated in vacuum to dryness.

### 4.3. Isolation of Allelochemicals from the Rhizosphere Soil

The residue of the rhizosphere soil was dissolved in water, then extracted by petroleum ether, chloroform, ethyl acetate, and n-butanol saturated with water, to finally obtain five fractions (Fr. 1–5). Fr. 1 was fractionated by MCI gel column chromatography eluting with methanol/water (3:7, 1:1, 7:3 and 9:1, *v*/*v*), and finally eluted with methanol to get compound **1** (3.6 mg) and compound **2** (12.4 mg), respectively. Fr. 2 was separated by PTLC (petroleum ether/EtOAc, 10:1, *v*/*v*) to yield compound **3** (4.9 mg), compound **4** (2.3 mg), and Fr. 2–3. Fr. 2–3 was subjected on silica gel CC with petroleum ether/EtOAc (3:1) in mobile phase to give compound **5** (3.5 mg). Fr. 3 was submitted to silica gel CC and eluting with chloroform/methanol (10:1, *v*/*v*) to obtain compound **6** (2.5 mg). Fr. 4 was eluted with chloroform/methanol (3:1, *v*/*v*) on silica gel column chromatography to obtain compound **7** (6.3 mg).

### 4.4. Plant Material and Growth Conditions

Seeds of *Lactuca sativa* L. were purchased from Gansu Academy of Agricultural Sciences. After soaked with 10% sodium hypochlorite for 10 min, the test seeds were washed with distilled sterile water 3 times, then germinated on filter paper in the dark at 25 °C for 1 day.

Healthy potato cultivar Long 7 with strong growth potential and no virus carrying was used for the germination treatment. After germination, the lateral or top buds were cut into a beaker with a scalpel and rinsed with flowing water for 6 h. These materials were soaked with 75% alcohol for 45 s. Then, the buds were sanitized in 0.1% mercuric chloride for 10 min, and washed with sterile water 5 times in order to thoroughly remove residual Hg^+^ from explant material. The medium for the tissue culture of potatoes was modified on MS medium containing MS medium, agar (7 g/L), sucrose (30 g/L), and 1.5 mg/L naphthaleneacetic acid (NAA). Finally, the tissue culture seedlings (about 1–2 cm length) were cut and inserted into the medium, and placed in a culture chamber for light culture (in the light for 14 h and in the dark for 10 h per day) at 25 °C.

### 4.5. Bioassays

*L. sativa* was selected as the receptor plant, and the allelopathic activities of the crude extract and compounds **1**–**7** were evaluated by a plate culture method. The crude extract and compounds **1**–**7** were dissolved in DMSO and diluted with distilled sterile water, with final concentrations of 50, 100, 200, and 400 μg/mL for the crude extract and 10, 25, 50, 100, and 200 μg/mL for compounds **1**–**7**, respectively. The concentration of DMSO should be ensured not to exceed 1% (*v*/*v*). The same ratio of DMSO was added into distilled sterile water as a control. After germination, the test seedlings were transferred to 6-well plates (Corning, Inc., Corning, NY, USA). 0.5 mL of the solutions was added to each of the wells, and three replicates (six seedlings for each replicate) were set for each treatment. Then, the six-well plates were placed in a constant temperature and humidity chamber in the dark at 25 °C for 2 days. The lengths of the roots and stems were measured and the relative length of the control was calculated.

Tissue culture seedlings of potato were used to evaluate the autotoxic activities of the crude extract and compounds **1**–**7**. The crude extract and compounds **1**–**7** were dissolved in DMSO and added into the medium, with final concentrations of 50, 100, 200, and 400 μg/mL for the crude extract and 10, 25, 50, 100, and 200 μg/mL for compounds **1**–**7**, respectively. The same ratio of DMSO was added into the medium as a control. The tissue culture seedlings with the same growing trend were transferred to the prepared treatment and control medium, and three replicates (six seedlings for each replicate) were set for each treatment. Then, the seedlings were cultured in the same conditions as above for 10 days. The height of the seedlings, the fresh weight of the leaves and stems, and the fresh weight of the roots were measured and the relative ratio of the control was calculated.

### 4.6. High-Performance Liquid Chromatography (HPLC) Analysis

The existence of the allelochemicals in the rhizosphere soil of potato was confirmed by high-performance liquid chromatography (HPLC), and the contents of the allelochemicals were quantified simultaneously. The optimum gradient of the mobile phase was: 20–100% A (A: methanol; B: water) at 0–30 min and 100% (A) at 30–45 min. The detection wavelength was selected as 240 nm for compounds **1**, **2**, and **4**, and 280 nm for compound **6**. The flow rate was 1 mL/min with an injection volume of 20 μL, and the column temperature was 35 °C. The existence of these compounds in the soil was confirmed by comparing the retention times of the standard compounds **1**, **2**, **4**, and **6** under the same chromatographic conditions. By using the linear regression method, the standard curves were achieved. The contents of allelochemicals in the rhizosphere soil of potato could be calculated by using the peak area and linear equations. The results are presented in Table 1.

### 4.7. Antifungal Activity

The antifungal activity of compounds **1**–**7** was evaluated against *Alternaria solani* Sorauer, *Botrytis cinerea* Persoon, *Fusarium solani* (Mart.) Sacc, *Fusarium oxysporum* Schlecht, *Colletotrichum coccodes* (Wall) Hughes, and *Verticillium dahlia* by a mycelium growth rate test with some modifications [46] using PDA medium. All the plant pathogens were from the CAS Key Laboratory of Chemistry of Northwestern Plant Resources, Lanzhou Institute of Chemical Physics, Chinese Academy of Sciences, Lanzhou, China. The strains were identified by Hui Jin and Xiaoyan Yang from Lanzhou Institute of Chemical Physics, Chinese Academy of Sciences, Lanzhou, China. Compounds **1**–**7** were dissolved in DMSO and added into the PDA medium with a final concentration of 200 μg/mL; the concentration of DMSO should be ensured not exceed 1% (*v*/*v*). 10 mL of the treatment and control medium were poured into each sterilized Petri dish (60 mm in diameter), and the fungi mycelia disk with a 5.0 mm diameter was placed in the center of the Petri dish and incubated at 25 °C. When the mycelium of the fungi reached the edges of the control plate, the mycelia diameter of each treatment was measured.

### 4.8. Statistical Analysis

All data were subject to an analysis of variance by SPSS 18. The significant differences between the treatment groups and the control group were calculated by the Fisher’s least significant difference (LSD) test and one-way analysis of variance (ANOVA). Statistical significance was accepted at * *p* < 0.05, ** *p* < 0.01. The relative ratio (percent) was determined by the formula [treated group/control group] × 100.

## 5. Conclusions

In summary, the isolated compounds from the rhizosphere soil of potato had a close relation to the allelopathy and autotoxicity. The accumulation of the allelochemicals could induce an imbalance of the microbial community in the potato replanted soil and result in replant failure during continuous cropping. These findings are helpful for understanding the allelopathic factors and mechanism that lead to replant failure in the cultivation of potato.

## Figures and Tables

**Table 1:** Regression equations, correlation coefficients, linear range, retention time, and contents for the four allelochemicals.

Compounds	Linear Equations ^a^	R^2^	Linear Range (ng/mL)	Retention Time (min)	Content (μg/g)
**1**	*y* = 0.0093*x* − 343.9	0.9995	2000.0–30,000.0	23.503	1.46
**2**	*y* = 0.0133*x* + 123.7	0.9998	2000.0–30,000.0	32.605	6.64
**4**	*y* = 0.0102*x* + 212.15	0.9999	500.0–30,000.0	10.687	0.35
**6**	*y* = 0.0152*x* + 518.21	0.9993	2000.0–30,000.0	13.900	0.57

^a^ *y* represents the peak area, *x* represents the concentration (ng/mL).

## Data Availability

Not applicable.

## Abbreviations

ANOVA—analysis of variance; CC—column chromatography; GC−MS—gas chromatography−mass spectrometry; LSD—least significant difference; MCI—gel, middle chromatogram isolated gel; PTLC—preparative thin-layer chromatography; TLC—Thin-layer chromatography.
